# The HLA landscape of Colombia: a high-resolution analysis of 11,576 blood donors

**DOI:** 10.3389/fimmu.2025.1736784

**Published:** 2025-12-22

**Authors:** Ana Luisa Muñoz, Daniel Uricoechea, Lina Andrea Gómez, Hernán Argote-Bérdugo, Johan Bula, María Alejandra Rueda, Miguel Germán Rueda, Ignacio Briceño

**Affiliations:** 1 Fundación Banco Nacional de Sangre Hemolife, Bogotá, Colombia; 2 Grupo de Investigación Genética Humana, Facultad de Medicina, Universidad La Sabana, Chía, Colombia; 3 Centro de Investigación Biomédica (CIBUS), Facultad de Medicina, Universidad de La Sabana, Chía, Colombia; 4 Banco Nacional de Sangre, Barranquilla, Colombia

**Keywords:** Admixed Latin American populations, carrier frequency, Colombian blood donors, high-resolution HLA typing, HLA diversity, population relationship, transfusion and transplantation medicine

## Abstract

**Background:**

The highly polymorphic Human Leukocyte Antigen (HLA) system is critical for adaptive immunity and determines compatibility in transfusion and transplantation medicine. Admixed Latin American populations, such as Colombia’s trihybrid population, possess unique HLA diversity that remains poorly characterized at high resolution.

**Methods:**

We performed high-resolution HLA typing on 11,576 Colombian blood donors from two distinct regions: the Andean and Caribbean. We analyzed allele and haplotype frequencies, Hardy-Weinberg equilibrium, linkage disequilibrium (LD), and compared genetic distances with global populations via PCA and UPGMA clustering.

**Results:**

We identified 565 alleles and 17,317 unique haplotypes, revealing extreme diversity. A few alleles dominated each locus, yet the top 20 haplotypes had a cumulative carrier frequency of only 3.17%, highlighting a fragmented haplotype landscape. Strong LD was observed between class I and II loci (HLA-A~DQB1), indicating conserved extended haplotypes. Genetically, the Colombian cohorts formed a tight cluster, showing closest affinity to Indigenous Chilean populations, suggesting a shared Andean background.

**Discussion:**

This study provides the first large-scale, high-resolution HLA map of Colombia, capturing the extensive immunogenetic diversity of an admixed Latin American population. Our findings are vital for improving transplant matching, understanding population-specific disease risks, and advancing equitable genomic medicine in the region.

## 1 Introduction

The human leukocyte antigen (HLA) system, located on chromosome 6, is encoded by more than 200 highly polymorphic genes and functions to present antigenic peptides to the immune system. This system is divided into class I (HLA-A, -B, and -C) and class II (HLA-DR, -DQ, and -DP) loci. It plays a critical role in inducing and regulating both innate and adaptive immunity, and is consequently central to transfusion and transplantation compatibility, infectious disease responses, and pharmacogenomics (1).

In transfusion medicine, the absence of routinely HLA-matched blood products can lead to HLA sensitization, a condition associated with complications such as platelet refractoriness, transfusion-related acute lung injury (TRALI), and an increased risk of rejection in future transplants (2). Although leukoreduction and irradiation can mitigate these risks, their implementation is often inconsistent, particularly in developing countries like Colombia. There, leukoreduction remains limited, and irradiation is primarily reserved for preventing transfusion-associated graft-versus-host disease in high-risk patients (e.g., transfusion-associated graft-versus-host disease) (3). Avoiding HLA sensitization is crucial in multiple clinical settings including pregnancy, autoimmune diseases, and recurrent transfusions. Consequently, improving HLA compatibility represents an imperative goal for transfusion safety.

Advances in high-resolution HLA typing, now a gold standard in precision medicine, have revealed extensive allelic diversity, underscoring the need for population-specific genetic profiling. Latin American populations, particularly those with trihybrid ancestry (Indigenous, European, and African), such as found in Colombia, exhibit unique HLA diversity that remains markedly underrepresented in global databases (4, 5). This gap perpetuates healthcare disparities by limiting equitable access to safe transfusions, transplantation, and personalized therapies. Although a previous study demonstrated HLA genetic heterogeneity within Colombia (6), its findings were constrained by a limited sample size.

In this context, blood donors represent an ideal cohort for advancing such HLA studies. This population is typically large, ethnically diverse, and composed of essentially healthy volunteers, from whom biological samples and data can be ethically and readily obtained. The resulting high-resolution HLA data are of direct importance to transfusion medicine, as they can inform strategies to mitigate the risks of HLA sensitization and its associated complications, discussed above.

Establishing a local HLA databases is essential to enhance transfusion safety by minimizing alloimmunization risks in multi-transfused patients, improve transplant outcomes through better donor-recipient matching, especially for underrepresented groups, advance personalized medicine by enabling pharmacogenomics studies, and research on immune responses to endemic or emerging pathogens, and expand anthropogenetic knowledge of Colombia’s population history and its impact on immunogenetic variation.

Here, we present a high-resolution analysis of HLA diversity from over 11,500 Colombian blood donors across two distinct regions (Andean and Caribbean). This dataset provides a population-level characterization of HLA allele carrier status, genotype distributions and linkage disequilibrium measures in a Colombian cohort for HLA-A, -B, -DRB1, -DQB1, and -DPB1. These metrics collectively support clinical and immunogenetic applications such as donor–recipient matching feasibility, haplotype-based registry optimization, and regional benchmarking of immunogenetic diversity.

This resource is poised to bridge critical equity gaps in Latin-American genomic medicine and strengthen public health infrastructure. Furthermore, it establishes a foundational dataset for future research on Colombian HLA diversity, the interplay between genetic ancestry and immunogenetic variation, HLA-pathogen interactions in endemic infections, and HLA- disease associations.

## 2 Materials/subjects and methods

### 2.1 Samples

The study subjects consisted of voluntary blood donors who met the national eligibility criteria, and provided informed consent specifically for HLA genotyping analysis, as approved by the Dexa Diab Scientific Research in Ethics Committee (Approval Codes: CE-CC-01522 and CE-CC-01555). All procedures were conducted in accordance with the Declaration of Helsinki and local regulations. A total of 11,576 donors were included, participants were recruited from Bogotá and the surrounding Andean region (n=10,447, 90.25%), and from Barranquilla and the surrounding Caribbean region (n=1,129, 9.75%). The cohort comprised 46.27% males and 53.73% females with ages ranging from 18 to 60 years. Buccal swab samples were collected, and subjected to high-resolution HLA typing for HLA-A, -B, -C, -DRB1, -DQB1, and -DPB1 loci using next-generation sequencing (NGS)-based methods.

### 2.2 High-resolution HLA genotyping and carrier frequency analysis in a cohort of Colombian blood donors

The analysis encompassed the following loci: HLA-A, -B, and -C (class I), and HLA-DRB1, -DQB1, and -DPB1 (class II). Alleles were coded according to the G-group nomenclature to group sequences based on functional equivalence.

We estimated the carrier frequency. Thus, for each HLA allele, we report three complementary metrics:

- The absolute number of positive individuals (carriers: individuals with ≥1 copy of the allele);
- The carrier frequency as a percentage (number of positive individuals/total cohort size) × 100; and
- The carrier frequency as a proportion (dimensionless), calculated as number of positive individuals/total cohort size.

These metrics reflect genotype-level presence—not allele copy number—. Carrier frequencies were calculated by direct counting using Arlequin software package (version 3.5.2.2), which also provided tools for estimating the Hardy-Weinberg equilibrium (HWE) using Fisher’s exact test and for evaluating linkage disequilibrium (LD) between allele pairs through 2x2 contingency tables. Extended haplotype frequencies for combinations (A~B~C~DQB1~DRB1~DPB1) were inferred using phased genotype data.

Subsequent data processing, including the filtration of haplotypes with extended frequencies below 1×10^-6^ and summary statistics, was conducted using Microsoft Excel.

To place the Colombian dataset within a broader genetic context, we compared it with regional and international populations, through Euclidean distance matrices calculated from carrier frequencies. Genetic relationships were assessed using several complementary approaches: Principal Component Analysis (PCA) and clustering based on the Unweighted Pair Group Method with Arithmetic Mean (UPGMA) were applied to assess genetic affinities among populations, with particular emphasis on relationships between the Colombian cohorts and Indigenous groups from Chile. Furthermore, we quantified genetic differentiation by calculating locus-specific fixation index (FST) values and performed a simplified Analysis of Molecular Variance (AMOVA) to partition genetic variance into within- and between-population components. Together, this multi-faceted analytical framework provided a robust and integrative assessment of immunogenetic structure and diversity in the Colombian population.

### 2.3 Data inclusion criteria

To ensure analytical accuracy and reliability of the analyses, a rigorous data cleaning protocol was implemented prior to statistical evaluation. This involved the removal of all genotypes with incomplete allele information, duplicated entries, and inconsistent haplotype configurations. The resulting curated dataset consisted exclusively of high-resolution, unambiguous HLA genotypes across all six loci (HLA-A, -B, -C, -DRB1, -DQB1, and -DPB1), providing a robust downstream analysis of allelic frequencies, haplotypes, and population structure.

### 2.4 Statistical analysis

HLA alleles were categorized using G-group nomenclature for all analyses. Carrier frequencies for HLA-A,-B,-C,-DRB1,-DQB1, and -DPB1 loci were calculated by direct counting using Arlequin software (version 3.5.2.2). Extended haplotype frequencies were calculated using Microsoft Excel for data processing. Haplotypes with an extended frequency below 1×10– (6) were not reported. Deviations from Hardy-Weinberg equilibrium (HWE) were tested for each locus using Fisher’s exact test, and linkage disequilibrium (LD) between allele pairs was estimated using Arlequin software (version 3.5.2.2). A p-value of < 0.05 was considered significant in determining deviations from HWE and assessing LD.

## 3 Results

### 3.1 Carrier frequency

#### 3.1.1 Class I allele frequency

High-resolution HLA typing of the 11,576 samples identified a total of 565 distinct alleles across the six loci. The distribution was as follows: 114 alleles for HLA-A, 183 for HLA-B, 78 for HLA-C, 88 for HLA-DRB1, 41 for HLA-DQB1, and 61 for HLA-DPB1. Carrier frequencies (number and percentage of positive individuals) for high-resolution HLA alleles (G-group level) are presented in **Table 1**.

**Table 1:** Genotype-based carrier frequencies (absolute and proportional) for Class I allele frequency in a Colombian sample.

HLA-A Allele	Number of positive individuals (N)	Carrier frequency (%)	Carrier frequency	HLA-B Allele	Number of positive individuals (N)	Carrier frequency (%)	Carrier frequency	HLA-C Allele	Number of positive individuals (N)	Carrier frequency (%)	Carrier frequency
24:02:01G	5023	21.63	0.43	35:43:01G	1990	8.57	0.17	04:01:01G	3617	15.63	0.31
02:01:01G	3690	15.89	0.32	40:02:01G	1856	7.99	0.16	01:02:01G	2523	10.91	0.22
01:01:01G	1451	6.25	0.13	44:03:01G	1361	5.86	0.12	07:02:01G	2426	10.49	0.21
03:01:01G	1246	5.37	0.11	07:02:01G	1282	5.52	0.11	03:04:01G	1895	8.19	0.16
29:02:01G	1079	4.65	0.09	51:01:01G	1266	5.45	0.11	07:01:01G	1796	7.76	0.16
68:01:02G	969	4.17	0.08	35:01:01G	1144	4.92	0.10	08:02:01G	1231	5.32	0.11
11:01:01G	952	4.1	0.08	14:02:01G	1051	4.52	0.09	16:01:01G	1229	5.31	0.11
31:01:02G	839	3.61	0.07	18:01:01G	836	3.6	0.07	06:02:01G	1180	5.1	0.10
02:22:01G	749	3.23	0.06	44:02:01G	784	3.38	0.07	05:01:01G	1105	4.78	0.10
23:01:01G	709	3.05	0.06	08:01:01G	720	3.1	0.06	15:02:01G	992	4.29	0.09
30:02:01G	575	2.48	0.05	39:05:01G	684	2.94	0.06	12:03:01G	955	4.13	0.08
26:01:01G	574	2.47	0.05	35:12:01G	676	2.91	0.06	03:05:01G	825	3.57	0.07
32:01:01G	497	2.14	0.04	38:01:01G	562	2.42	0.05	02:02:02G	524	2.26	0.05
68:02:01G	487	2.1	0.04	49:01:01G	522	2.25	0.05	03:03:01G	378	1.63	0.03
2:13	409	1.76	0.04	53:01:01G	479	2.06	0.04	17:01:01G	372	1.61	0.03
33:01:01G	393	1.69	0.03	48:01:01G	435	1.87	0.04	08:01:01G	352	1.52	0.03
30:01:01G	378	1.63	0.03	15:01:01G	414	1.78	0.04	14:02:01G	248	1.07	0.02
24:14:01G	374	1.61	0.03	50:01:01G	400	1.72	0.03	02:10:01G	232	1	0.02
02:05:01G	369	1.59	0.03	57:01:01G	329	1.42	0.03	12:02:01G	196	0.85	0.02
25:01:01G	211	0.91	0.02	45:01:01G	312	1.34	0.03	07:04:01G	135	0.58	0.01

HLA-A locus:

The most frequent allele at this locus was A*24:02:01G, with a carrier frequency of 21.63% (n = 5,023). Followed by A*02:01:01G with a carrier frequency of 15.89%, (n = 3,690). While A*02:01:01G is relatively common, it is less prevalent than A*24:02:01G. Other alleles with notable carrier frequencies included A*01:01:01G (6.25%) and A*03.01:01G (5.37%). The least frequent allele observed was A*23:01:01G, which appears in 709 samples and represented a carrier frequency of 3.05% (**Table 1**).

The carrier frequency distribution at the HLA-A locus is characterized by the dominance of a few common alleles alongside a long tail of lower-frequency alleles, which collectively contribute substantial diversity to the HLA-A locus.

HLA-B Locus:

The HLA-B locus exhibited the greatest allelic diversity among the Class I genes, with a total of 183 distinct alleles identified. The most frequent allele was B*35:43:01G, representing 8.57% of the sample (n = 1,990), closely followed by B*40:02:01G at 7.99% (n = 1,856). A second tier of moderately frequent alleles included B*44:03:01G (5.86%) and B*07:02:01G (5.52%). In contrast B*08:01:01G, was observed as the least frequent allele at 3.10% (n = 720) (**Table 1**). This profile confirms a characteristic pattern for HLA loci: a small set of common alleles defines the population background, while a substantial number of lower-frequency alleles, such as B*08:01:01G, are responsible for the majority of the locus’s high heterogeneity.

HLA-C Locus:

At the HLA-C locus, the most frequent allele was C*04:01:01G, with a carrier frequency of 15.82% (n = 3,617). The second most common allele was C*01:02:01G (11.03%, n = 2,523), Furthermore, C*07:02:01G and C*03:04:01G alleles showed moderate carrier frequencies, representing 10.59% and 8.28%, respectively. The least frequent allele observed was C*02:02:01G, representing 2.79% of the sample (n = 637) (**Table 1**). Mirroring the pattern seen at HLA-A and -B, the HLA-C locus is characterized by a subset of predominant alleles that form the genetic backbone of the population, complemented by a spectrum of lower-frequency alleles that are the primary drivers of its overall diversity.

#### 3.1.2 Class II allele carrier frequency

High-resolution typing of the 11,576 samples for Class II loci identified 190 distinct alleles (88 for HLA-DRB1, 41 for HLA-DQB1, and 61 for HLA-DPB1). Carrier frequencies for high-resolution Class II HLA alleles (G-group level) are presented in **Table 2**.

**Table 2:** Genotype-based carrier frequencies (absolute and proportional) for Class II allele frequency in a Colombian sample.

HLA-DRB1	Number of positive individuals (N)	Carrier frequency (%)	Carrier frequency	HLA-DQB1	Number of positive individuals (N)	Carrier frequency (%)	Carrier frequency	HLA-DPB1	Number of positive individuals (N)	Carrier frequency (%)	Carrier frequency
04:07:01G	2842	12.25	0.25	03:02:01G	4965	21.42	0.43	04:02:01G	5894	22.39	0.51
07:01:01G	2269	9.78	0.20	03:01:01G	4488	19.36	0.39	04:01:01G	5785	21.98	0.50
08:02:01G	1541	6.64	0.13	02:01:01G	3544	15.29	0.31	02:01:02G	2570	9.77	0.22
15:01:01G	1359	5.86	0.12	04:02:01G	2593	11.19	0.22	14:01:01G	2538	9.64	0.22
03:01:01G	1358	5.85	0.12	05:01:01G	2402	10.36	0.21	03:01:01G	1381	5.24	0.12
13:01:01G	1165	5.02	0.10	06:02:01G	1677	7.23	0.14	01:01:01G	1053	4	0.09
14:02:01G	1003	4.32	0.09	06:03:01G	1141	4.92	0.10	17:01:01G	659	2.5	0.06
16:02:01G	924	3.98	0.08	03:03:02G	558	2.41	0.05	11:01:01G	616	2.34	0.05
04:04:01G	905	3.9	0.08	06:04:01G	506	2.18	0.04	13:01:01G	551	2.09	0.05
13:02:01G	815	3.51	0.07	05:03:01G	439	1.89	0.04	05:01:01G	507	1.93	0.04
01:01:01G	808	3.48	0.07	05:02:01G	283	1.22	0.02	10:01:01G	322	1.22	0.03
01:02:01G	790	3.41	0.07	06:09:01G	245	1.06	0.02	02:02:01G	263	1	0.02
11:01:01G	678	2.92	0.06	06:01:01G	184	0.79	0.02	06:01:01G	148	0.56	0.01
4:05:04	446	1.92	0.04	03:04:01G	42	0.18	0.00	01:01:02G	105	0.4	0.01
11:04:01G	443	1.91	0.04	3:02:03	32	0.14	0.00	09:01:01G	100	0.38	0.01

HLA-DRB1 Locus:

The carrier frequency distribution for HLA-DRB1 was dominated by DRB1*04:07:01G with a carrier frequency of 12.25%, (n = 2,842), and DRB1*07:01:01G (9.78**%** n = 2,269). A secondary group of alleles with moderate frequencies included DRB1*08:02:01G (6.64%) and DRB1*15:01:01G (5.86%). The lower end of the carrier frequency spectrum was marked by alleles such as DRB1*13:02:01G (3.51%, n = 815), making up 3.51% of the total HLA-DRB1 samples. This architecture—a small set of common alleles underpinning the population profile, augmented by a long tail of less frequent variants—confirms that the HLA-DRB1 locus is a major contributor to the overall immunogenetic diversity in the Colombian cohort.

HLA-DQB1 Locus:

The HLA-DQB1 locus was characterized by a high-frequency dominance of two primary alleles. The most frequent allele for this locus was DQB1*03:02:01G, with a carrier frequency of 21.42% (n = 4,965). It was closely followed by DQB1*03:01:01G at 19.36**%** (n=4,488), indicating these two variants alone account for over 40% of the allele pool. Other common alleles included DQB1*02:01:01G (15.29%) and DQB1*04:02:01G (11.19%). In contrast, DQB1*05:03:01G was the least frequent allele observed, representing 1.89% of the sample (n = 439) (**Table 2**). This distribution underscores a pronounced concentration of frequency in a few common alleles, while the presence of a spectrum of rarer alleles, like DQB1*05:03:01G, maintains the locus’s significant genetic heterogeneity.

HLA-DPB1 Locus:

A remarkable finding at the HLA-DPB-1 locus was the pronounced dominance of two closely related alleles DPB1*04:02:01G (22.39%, n = 5,894) and DPB1*04:01:01G (21.98%, n = 5,785). Together, these two variants constituted nearly 45% of the entire allele pool. A second tier of moderately frequent alleles include DPB1*02:01:02G (9.77%) and DPB1*14:01:01G (9.64%). In contrast, the least frequent allele observed was DPB1*05:01:01G, representing just 1.93% of the sample (n = 507) (**Table 2**). This variability highlights the genetic complexity within this locus, with both highly recurrent and less frequent alleles contributing to the allele pool.

The analysis of HLA class II loci demonstrate a characteristic architecture of high-frequency dominance alongside considerable underlying diversity. This pattern was most pronounced at the HLA-DQB1 locus, where two alleles DQB1*03.02:01G and DQB1*03:01:01G make up a substantial proportion of all samples, constituted over 40% of the allele pool. A similar, though less extreme, concentration was observed for DRB1*04:07:01G and DRB1*07:01:01G. Notably, the HLA-DPB1 locus exhibited the most striking dominance, with the DPB1*04:02:01G and DPB1*04:01:01G alleles accounting for nearly 45% of all occurrences. In each case, this core of prevalent alleles is complemented by a long tail of lower-frequency variants that are the primary drivers of the high heterogeneity observed. This consistent immunogenetic structure—a foundation of common alleles defining the population profile, enriched by a spectrum of rare alleles—highlights the critical balance between selective pressures and maintained diversity that is central to adaptive immune function.

**Figure 1:**
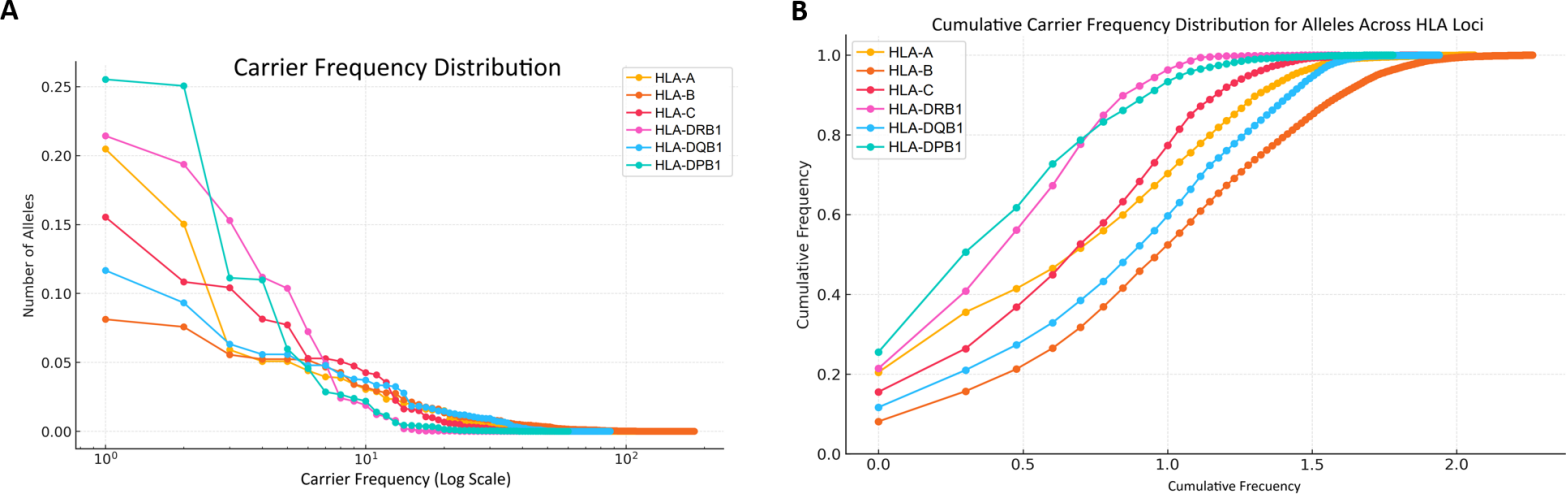
**(A)** Carrier frequency distribution across HLA systems (A, B, C, DRB1, DQB1, and DPB1). **(B)** Cumulative carrier frequency distribution plot for the alleles across each HLA locus (A, B, C, DRB1, DQB1, and DPB1).

Allele frequencies were calculated by direct counting, as mentioned above. For graphical representation of the population distribution, we plotted the carrier frequency for each allele, defined as the proportion of individuals in the cohort carrying at least one copy of that specific allele. So, Carrier frequencies of HLA alleles at each locus, ranked in descending order, are plotted in **Figure 1A**. A logarithmic y-axis was used to effectively visualize the wide range of carrier frequencies, from the most common to the rarest alleles. The normalized frequency curves reveal distinct architectural patterns across the loci. HLA-DPB1 is the most constrained, characterized by the highest frequencies for its dominant alleles, suggesting a strong selective pressures for conserved immune functions. In stark contrast, the classical Class I loci (HLA-A, -B, and -C) exhibit flatter, more parallel distributions, indicative of a broader array of alleles at moderate frequencies, reflecting a history of balancing selection that maintains high heterogeneity to combat a diverse pathogen landscape. The Class II loci HLA-DQB1 and HLA-DRB1 demonstrate an intermediate architecture, with a steep initial decline in frequency for the most common alleles, transitioning into a long tail of low-frequency variants. Thus, the detailed view of carrier frequencies, focusing on carrier frequencies up to 101.510^{1.5}101.5 (**Figure 1A**) provides additional insights into the systems’ diversity. This has significant implications for population genetics and immunogenetics. These fundamental differences in evolutionary trajectory have direct translational implications: a constrained locus like HLA-DPB1 simplifies population-level screening for transplantation, whereas the hyper-diversity of HLA-B is critical for understanding population-specific disease associations and the variability of adaptive immune responses.

Furthermore, the cumulative carrier frequency distributions for all six loci (HLA-A*, -B –C, -DRB1, -DQB1 and *-DPB1) are presented in **Figure 1B**. The x-axis is on a logarithmic scale, and the cumulative carrier frequency is shown on the y-axis. A curve that approaches the upper-left corner of the plot indicates a more homogeneous locus, where a small number of alleles account for a large portion of the genetic diversity. The relative positions of the curves provide an immediate, quantitative comparison of heterogeneity across the HLA system. This visualization highlights the cumulative contribution of alleles in each locus (A, B, C, DRB1, DQB1, DPB1), allowing a detailed comparison of genetic diversity across loci.

The logarithmic representation of allele rank underscores the contrast between loci with dominant alleles (e.g., HLA-DPB1) and those with a more even distribution (e.g., HLA-B and HLA-C). It effectively demonstrates how carrier frequency drops steeply for dominant loci and more gradually for diverse loci.

### 3.2 Expanded haplotype frequencies

**Figure 2:**
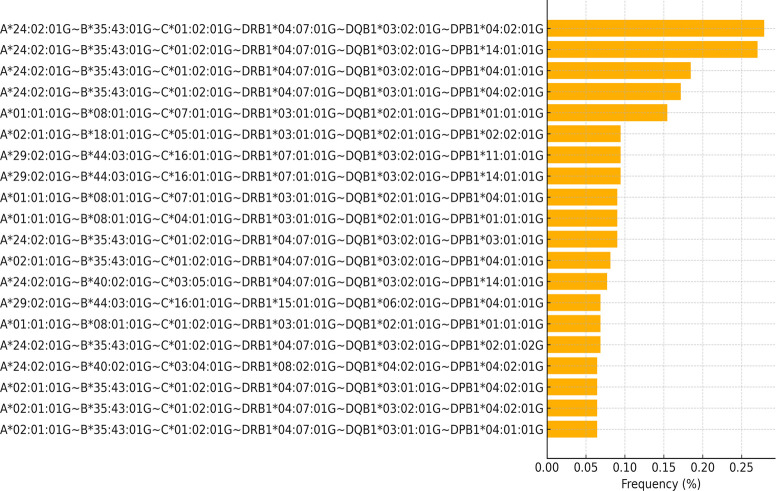
Top 20 six-locus HLA haplotype carrier frequency in a Colombian population.

A total of 17,317 unique six-locus haplotypes (HLA –A*, -B, -C, -DRB1, -DQB1, and *-DPB1) were identified, highlighting the exceptional haplotypic diversity within this admixed Colombian population. For high-resolution analysis, we focused on haplotypes with complete, unambiguous information across all six loci, yielding 18-allele combinations (three components per locus: field1, field2, suffix) per haplotype. The frequencies expanded of the top seven of these haplotypes are shown in **Figure 2**. Strikingly, the 20 most frequent combinations collectively accounted for only 3.17% of all observed genotypes, underscoring a highly fragmented haplotype landscape where no single haplotype is predominant.

The most frequent individual haplotype, present in 65 individuals (0.28%), was A*24:02:01G~B*35:43:01G~C*01:02:01G~DRB1*04:07:01G~DQB1*03:02:01G~DPB1*04:02:01G. Notably, this combination integrates the most frequent allele from four of the six loci (A, B, DRB1, DQB1), forming a candidate population-specific haplotype block that aligns with prevalent alleles reported in other urban admixed Latin American cohorts. Furthermore, we observed the recurrence of highly similar haplotypes among the top entries, often differing only in a single allelic subtype (e.g., C04:01:01G vs. C01:02:01G), suggesting patterns of strong, extended linkage disequilibrium maintained within this population.

### 3.3 Hardy-Weinberg equilibrium test

We evaluated Hardy-Weinberg equilibrium (HWE) at each of the six classical HLA loci using observed and expected homozygosity frequencies and a chi-square test for deviation from equilibrium. The observed homozygosity values ranged from 10.27% (HLA-B) to 27.82% (HLA-DQB1), closely mirroring the expected ranged of 9.50% to 27.20% for the same loci, respectively.

No locus showed statistically significant deviations from HWE at the 0.05 level, with all p-values ranging from 0.20 to 0.69. This was further supported by the Warning statistic (Wn), defined as the difference between observed and expected homozygosity, which remained below 0.01 across all loci, indicating only negligible deviations from Hardy–Weinberg expectations.

These results suggest that the genotypic distributions at all six HLA loci are in Hardy-Weinberg equilibrium. This finding supports the quality of the genotype data and suggests that the sampled population is large, randomly mating, and free of significant genotyping artifacts for these loci. A complete summary of the HWE analysis, including homozygosity frequencies, Wn, and p-values, is provided in **Table 3**.

**Table 3:** Hardy-weinberg equilibrium in a Colombian sample.

Locus	Obs. Homozygosity	Exp. Homozygosity	Wn	Chi2 p-value
HLA-A	0.1558	0.1465	0.0093	0.20896
HLA-B	0.1027	0.095	0.0078	0.42474
HLA-C	0.1172	0.1142	0.0031	0.69836
HLA-DRB1	0.1296	0.122	0.0076	0.57999
HLA-DQB1	0.2782	0.272	0.0061	0.46938
HLA-DPB1	0.2981	0.2929	0.0052	0.8875

### 3.4 Linkage disequilibrium

**Figure 3:**
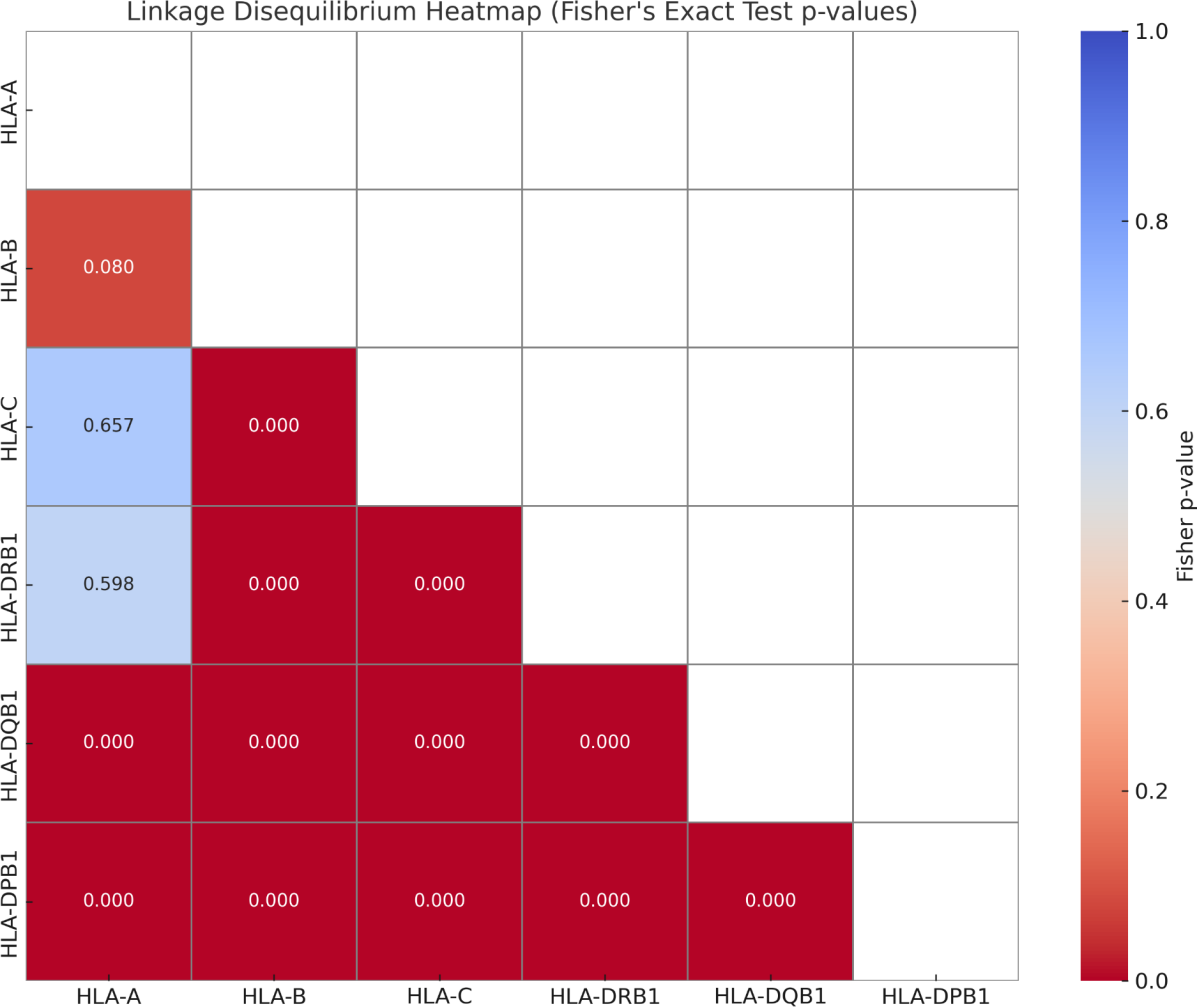
Heat map of Fisher’s exact test p-values for HLA-A~B~C~DRB1~DQA1~DQB1 linkage disequilibrium (LD). Lower p-values (red) indicate stronger LD. Significance for LD was calculated by performing a likelihood-ratio test using Arlequin software; LD levels are expressed as D’ value for p ≤0.01.

Linkage disequilibrium (LD) was assessed between all pairwise combinations of the six HLA loci using Fisher’s exact test. For each locus pair, a 2x2 contingency table was constructed based on the phased co-occurrence of their two most frequent alleles. The resulting p-values for all pairs are summarized in **Table 3**, and visualized in a heat map in **Figure 3**.

The analysis revealed strong, highly significant LD (p < 0.00001) between HLA-A and the Class I loci HLA-C and the Class II loci HLA-DRB-1. This indicates the presence of conserved extended haplotypes spanning the Class I and Class II regions, suggesting these combinations have been maintained through generations in the population. On the other hand, no significant LD association was detected between HLA-A and the other Class II loci (HLA-DPB1 and HLA-DQB1), suggesting a history of frequent recombination and allelic shuffling within the central Major Histocompatibility Complex (MHC).

These findings are consistent with established models of the MHC, where specific, long-range haplotypes are often conserved in populations, particularly the well-documented DRB1-DQB1 block. The identification of a stable HLA-A~DQB1/DPB1 association in this Colombian cohort underscores the population-specific architecture of these extended haplotypes. This has direct implications for improving the efficiency of donor recruitment strategies in transplantation and for refining association studies in population genetics.

### 3.5 Carrier frequencies and population comparison

We conducted a comparative analysis of high-resolution HLA carrier frequencies between two distinct Colombian subpopulations: the Andean region, characterized by high-altitude adaptation and predominantly European/Indigenous ancestry) and the Caribbean region, a coastal area with stronger African admixture.

**Figure 4:**
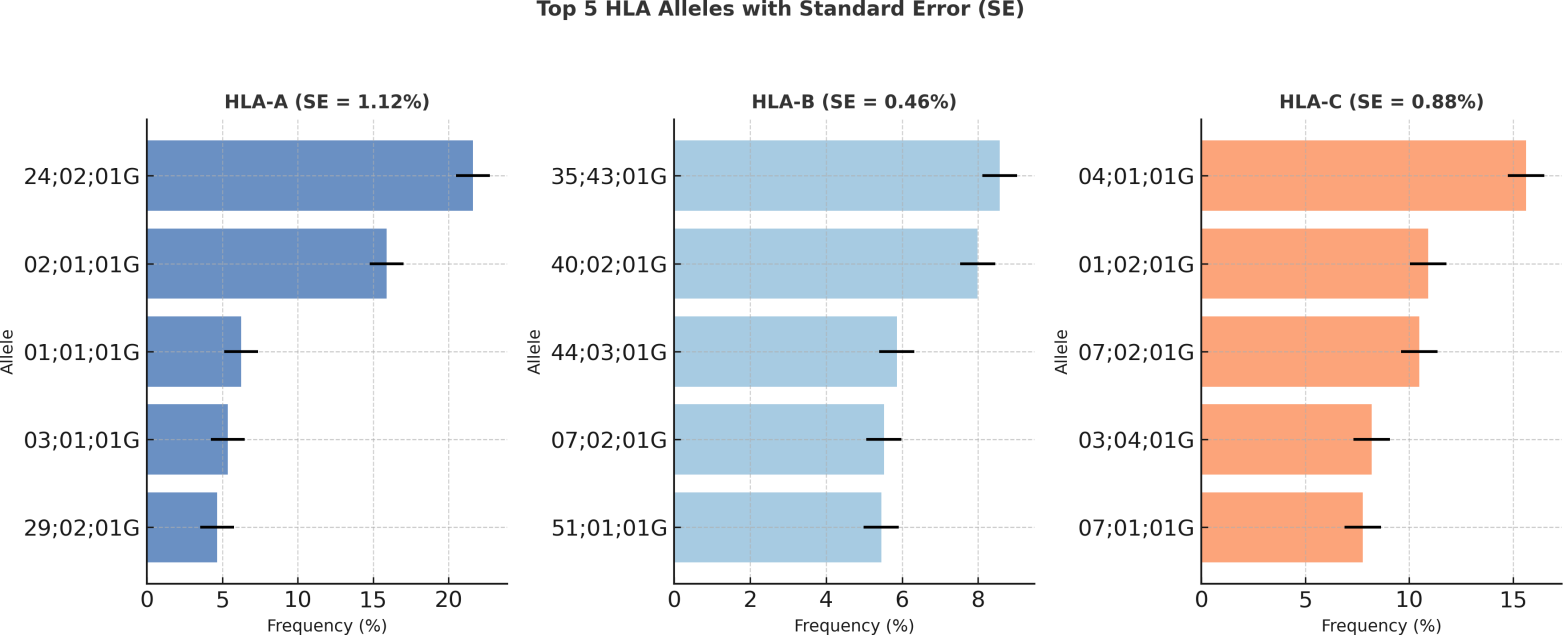
Carrier allelic frequencies and population comparison.

Carrier frequencies were calculated for each of the six HLA loci (HLA-A, HLA-B, HLA-C, HLA-DRB1, HLA-DQB1, and HLA-DPB1). The top five most frequent alleles at each locus are detailed in **Figure 4**. The allelic profiles of both regions are dominated by variants previously established as common in Colombia, including A*02, B*35, C*07, DRB1*04, DQB1*03, and DPB1*04. To assess whether allelic distributions differed significantly from those observed in reference populations, comparative analyses were performed using Fisher’s exact test, considering results statistically significant at p < 0.05.

Comparative data from our recent analysis of two Colombian departments, Atlántico (Caribbean) and Cundinamarca (Andean), confirms the prevalence of these same alleles. For instance, *A*24* consistently exceeds a 24% frequency in both populations, and *B*35*, and *DRB1*04* consistently rank among the dominant alleles. Despite this shared genetic background we identified marked inter-regional differences in allele distribution at specific loci, particularly HLA-B and HLA-DQB1, revealing subtle population substructure. This substructure reflects the distinct historical demographic and admixture influences that have shaped each region.

Furthermore, quantitative analysis of genetic differentiation yielded low fixation index (FST) values across all loci (FST < 0.01), supporting the hypothesis of substantial shared ancestry and significant gene flow between regional populations. This was corroborated by an AMOVA, which confirmed that the vast majority of genetic variance resides within, rather than between, these populations. These findings paint a coherent picture of a genetically cohesive national population, shaped by a common history yet fine-tuned by region-specific evolutionary pressures. This nuanced understanding is critical for refining transplant matching strategies and for conducting well-controlled disease association studies within Colombia.

### 3.6 Genetic affinities of the Colombian samples based on HLA loci

In the comparative analysis of carrier frequencies at HLA-A, HLA-B, HLA-C, HLA-DRB1, HLA-DQB1, and HLA-DPB1 loci between our two Colombian cohorts—the Andean region (CUN, n=10,447) and the Caribbean region (ALT, n=1,129)- and a range of international and Chilean reference populations from a stem cell donor study (7). The reference populations, denoted by standard codes (e.g., AR: Argentinian, BOL: Bolivian, BR: Brazilian, CO: Colombian, D: German, E: Spanish, ENG: English, F: French, HISP: US Hispanics, I: Italian, IND: Indian, IRL: Irish, MEX: Mexican, P:Portuguese, PE: Peruvian, PL: Polish, TR: Turkish, VE: Venezuelan, and also Northern Chilean Interior (CL-NI) and Mapuche (CL-MP) groups.

**Figure 5:**
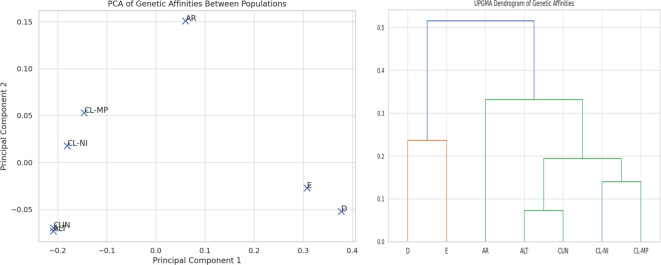
Genetic affinities of Colombian samples based on HLA loci, demonstrated by multidimensional scaling (PCA) and hierarchical clustering (UPGMA).

For this analysis allele frequencies from ALT and CUN were curated and integrated into a comprehensive dataset with international and Chilean references: Northern Chile Interior (CL-NI) and Mapuche (CL-MP). All alleles were validated per locus. Euclidean distance matrices were calculated for each locus and averaged. Multidimensional scaling (PCA) and hierarchical clustering (UPGMA) were applied. The Caribbean (ALT) and Andean (CUN) populations form a tight genetic cluster with a minimal average pairwise distance (0.051), underscoring their strong genetic similarity. Both Colombian groups exhibit notable proximity to Northern Chilean Interior (CL-NI) populations (ALT-CL-NI: 0.111; CUN-CL-NI: 0.109) and Mapuche groups (CL-MP) (ALT-CL-MP: 0.138; CUN-CL-MP: 0.135), suggesting shared ancestral or demographic histories, how is shown in **Figure 5**.

This clustering is visually confirmed in the PCA plot, where ALT and CUN form a distinct, cohesive cluster that is closely related to the Chilean populations (**Figure 5**). Finally, UPGMA dendrogram further corroborates this structure, Colombian and Chilean populations together on a primary branch that diverges separately from European and Asian clusters.

Collectively, these results suggest that two Colombian population analyzed shared a strong, common South American genetic background, likely shaped by deep historical connections such as pre-Columbian migrations and trade networks across the Andes. This shared ancestry may have facilitated parallel immunological adaptations to similar environmental pressures in this region.

## 4 Discussion

It is well known that the HLA system plays a decisive role in clinical practice. Its extensive polymorphism and immunogenic poses significant challenges in transfusion and transplantation medicine. Currently, a high percentage of patients, including multi-transfused individuals, receive HLA-mismatched and even non-leukoreduced blood components, which can trigger the production of cytotoxic anti-HLA antibodies and, therefore, the alloimmunization of these patients (8). This alloimmunization can lead to serious complications such as platelet refractoriness, transfusion-related acute lung injury (TRALI), and in rare cases, non-hemolytic febrile transfusion reaction. Furthermore, immunocompetent cells in the blood component could recognize receptor cells’ histocompatibility antigens and generate a transfusion-associated graft-versus-host disease (8). The large, diverse, and readily accessible cohort of volunteer blood donors thus represents an invaluable resource for building the immunogenetic knowledge base needed to address these clinical challenges.

Colombia’s trihybrid ancestry (European, African, and Indigenous American) has generated a unique HLA landscape, one that remains poorly characterized at high resolution. Our study addresses this critical gap by presenting the first high-resolution of HLA allele and haplotype frequency analysis in a large-scale Colombian cohort *(*n = 11,576 blood donors), covering six loci (HLA-A, -B, -C, -DRB1, -DQB1, -DPB1). This represents one of the most comprehensive immunogenetic datasets for a Latin American population to date. By comparing two geographically and ancestrally distinct subpopulations: the high-altitude Andean region (predominantly Indigenous American/European ancestry, >2–000 meters above sea level) and the coastal Caribbean region (with stronger African admixture), we reveal a genetic complexity that directly reflects the nation’s rich demographic history, characterized by admixture among Indigenous, European, and African ancestral sources.

Our analysis identified 565 distinct allelic groups for HLA A*, B*, C*, DRB1*, DQB1* and DPB1* of the about 35,000 reported worldwide (9). The results reveal a high degree of genetic complexity shaped by the country’s rich demographic history, characterized by admixture among Indigenous, European, and African ancestral sources. *S*imilar to high-resolution HLA studies in Latin America that have revealed high levels of diversity based on number of HLA alleles for each HLA loci, as well as high heterozygosity in Brazilian (10) and Mexican populations (11).

In class I, the HLA-A, -B, and -C loci demonstrated considerable allelic diversity, particularly HLA-B, exhibited considerable polymorphism, with prevalent alleles such as A*24:02:01G (21.63%), B*35:43:01G (8.57%), and C*04:01:01G (15.63%), supporting findings from previous Colombian studies (6, 12) and other admixed populations (1), while also suggesting the persistence of conserved haplotypes. Among class II loci, DRB1 and DQB1 displayed moderate-to-high polymorphism, with DRB1*04:07:01G (12.25%) and DQB1*03:02:01G, being the most common, frequencies also similar to those reported in Colombian studies (6, 12). Interestingly, DPB1 displayed a more constrained diversity, with two alleles—DPB1*04:02:01G *and* DPB1*04:01:01G—accounting for nearly 44% of all observations. A pattern also noted in other global populations (Chinese, Caucasians and Saudi) (1). These results underscores the necessity for local genetic studies, as HLA frequencies can differ significantly, even among subgroups within an admixed nation like Colombia.

The extreme haplotype diversity we observed—17,317 distinct six-locus combinations— these results are aligned with prior studies in admixed Latin American populations, where high haplotype diversity and low frequency of top haplotypes (<5%) are consistently reported (13, 14). The top 20 haplotypes had a cumulative frequency of only 3.17%, a pattern mirroring findings in Mexican Mestizos and Brazilians (10, 15). This reflects a high degree of heterogeneity, with no single haplotype dominating the distribution, and evidence that Interbreeding increases rare haplotypes, underscoring the impact of trihybrid ancestry (Indigenous, European, African) on HLA architecture. This heterogeneity, driven by trihybrid ancestry, complicates donor-recipient matching but profoundly enriches the region-specific immunogenetic dataset, representing a critical step toward reducing disparities in genomic research coverage (16), thus our results represent meaningful progress in bridging population disparities in genomic research coverage.

In contrast, HLA profiles among Colombian Amerindian groups, show a very low allele diversity. Therefore, some alleles are in high frequency as observed in class II DRB1, where alleles *0403, 0407 and *0411 are common (17). Several alleles are shared with Asian and Pacific Islander populations (18). Additionally, research on Afro-American populations, such as those in San Basilio de Palenque, reveals a unique genetic heritage reflective of this community’s ancestry (19), highlight the distinct HLA gene frequencies in this semi-isolated population, emphasizing the need for population-specific HLA databases, particularly in admixed Latin American populations.

Our population genetics analyses provide further insights. The absence of significant deviation from Hardy-Weinberg equilibrium across all loci indicates a stable, randomly mating population sample (20, 21). Furthermore, we detected strong linkage disequilibrium (LD), not only within but also *between* class I and class II loci (e.g., HLA-A with DQB1). This points to the preservation of extended haplotypes, likely maintained by balancing selection (22). These findings are consistent with prior reports of conserved HLA haplotype blocks in other admixed populations (13, 14).

In addition to carrier and expanded haplotype frequency analyses, this study conducted a comprehensive evaluation of linkage disequilibrium (LD) across the six classical HLA loci. Strong LD signals were observed particularly between class II loci and between class I and class II regions, most notably between HLA-A and HLA-DQB1, as well as HLA-B and HLA-DPB1, suggesting the presence of extended haplotypes likely maintained by selective pressures. These non-random associations support the hypothesis of conserved haplotypic structures within the Colombian population. Furthermore, population-level comparisons performed using Euclidean distance matrices, principal component analysis (PCA), and UPGMA clustering, revealed close genetic affinities between Colombian samples and Indigenous Chilean populations such as the Mapuche and Northern Interior groups (7) (**Figure 4**). Shared haplotypes between Peruvian, Bolivian, and Chilean Indigenous groups point to a continuum genetic exchange extending northward to Colombia (23), indicating that there is an ancient population continuity and later divergence, thus this close genetic proximity suggesting pre-Columbian migrations along the Andes. These patterns are consistent with a shared Andean genetic background shaped by ancient population movements (24, 25) and reflect both ancient population movements and more recent admixture events (26). This admixed background creates population-specific disease risks and pharmacogenomic profiles (27–29), but at the same time, unique HLA haplotypes from admixture complicate, for example, efforts in transplant matching and other areas of precision medicine, thereby necessitating tailored clinical approaches.

One limitation of this study is that, although it provides a comprehensive characterization of known allelic variants in HLA class I and II genes, systematic discovery of novel alleles was not feasible. Genotyping was performed by a third-party service, and only final consensus calls—not raw sequencing data—were accessible, per contractual agreement, to the authors. Consequently, the absence of reported novel alleles reflects methodological constraints rather than definitive allelic exhaustiveness in the population. Future studies will be essential to identify rare or population-specific variants with potential functional relevance to immune response and disease susceptibility.

In summary, this high-resolution HLA characterization of the Colombian population, particularly in Bogotá, provides an essential foundation for advancing public health and serves as an essential resource for future clinical and anthropological research. The findings underscore the critical need for localized genomic research to capture the unique immunogenetic landscape of underrepresented populations. Genomic research is essential for optimizing diagnostics, therapeutics, and ultimately, for achieving health equity in transfusion medicine, transplantation, and beyond. This dataset constitutes a national immunogenetic reference for Colombia and key contribution to Latin American population genomics.

## Data Availability

The raw data supporting the conclusions of this article will be made available by the authors, without undue reservation, upon a reasonable request.
